# A Lysine at the C-Terminus of an Odorant-Binding Protein is Involved in Binding Aldehyde Pheromone Components in Two Helicoverpa Species

**DOI:** 10.1371/journal.pone.0055132

**Published:** 2013-01-25

**Authors:** Ya-Lan Sun, Ling-Qiao Huang, Paolo Pelosi, Chen-Zhu Wang

**Affiliations:** State Key Laboratory of Integrated Management of Pest Insects and Rodents, Institute of Zoology, Chinese Academy of Sciences, Beijing, China; University of the Witwatersrand, South Africa

## Abstract

Odorant-binding proteins (OBPs) are soluble proteins, whose role in olfaction of insects is being recognized as more and more important. We have cloned, expressed and purified an OBP (HarmOBP7) from the antennae of the moth *Helicoverpa armigera*. Western blot experiments indicate specific expression of this protein in the antennae of adults. HarmOBP7 binds both pheromone components Z-11-hexadecenal and Z-9-hexadecenal with good affinity. We have also performed a series of binding experiments with linear aldehydes, alcohols and esters, as well as with other compounds and found a requirement of medium size for best affinity. The affinity of OBP7, as well as that of a mutant lacking the last 6 residues does not substantially decrease in acidic conditions, but increases at basic pH values with no significant differences between wild-type and mutant. Binding to both pheromone components, instead, is negatively affected by the lack of the C-terminus. A second mutant, where one of the three lysine residues in the C-terminus (Lys123) was replaced by methionine showed reduced affinity to both pheromone components, as well as to their analogues, thus indicating that Lys123 is involved in binding these compounds, likely forming hydrogen bonds with the functional groups of the ligands.

## Introduction

Odorant-binding proteins (OBPs) are soluble polypeptides present at the interface between the external environment and chemoreceptors in most animal species. Despite their common name, OBPs of insects and those of vertebrates are completely different in structures, a compact arrangement of α-helical domains in the first case [1], a β-barrel scaffolding in the second [2], [3]. However, similar functions have been suggested for the two classes of proteins [4], [5]. A large amount of structural and functional data, accumulated in more than 30 years since their discovery [6]–[10] have not afforded clear cues on their mode of action and their specific role in chemoreception. Only in the last few years some evidence has been produced on the requirement of OBPs for odour and pheromone chemoreception in insects and models based on experimental evidence have been proposed. In the moth *Antheraea polyphemus* the response of olfactory receptors to pheromones becomes much more sensitive and specific in the presence of the appropriate Pheromone-binding Protein (PBP) [11]. In *Drosophila melanogaster*, when the gene for LUSH, an OBP binding the male sex pheromone vaccenyl acetate, was knocked-out, the response to the pheromone was abolished, but could be fully restored when the gene was re-inserted [12]. Evidence that OBPs are involved not just in detection, but also in discrimination of olfactory stimuli was provided using behaviour studies. A first paper reported that the specific attraction of *Drosophila sechellia* to the fatty acids of its oviposition plant (that are repellents for other *Drosophila* species) can be linked to two OBPs, namely 57d and 57e. Exchanging the relative genes between *D. sechellia* and other *Drosophila* species produced a drastic change of the flies’ behaviour [13]. Another study measured the behaviour to several pure odorants of a number of *D. melanogaster* strains, each defective in a single specific OBP. Each of the mutant strains exhibited a completely different spectrum of response to the same set of odorants, thus showing that even the lack of a single OBP can markedly affect the performance of the olfactory system [14]. Finally, using ligand-binding studies and behavioural experiments, the repellent effect of the aphid alarm pheromone (*E*)-β-farnesene was significantly linked to a specific pattern of binding involving OBP3 and OBP7 and excluding other OBPs in two species of aphids [15], [16].

The mode of action of OBPs has been addressed by structural studies at the molecular level. First, it was observed that the pheromone-binding protein (PBP1) of *Bombyx mori* undergoes a major conformational change at acidic pH, where the C-terminal segment, unstructured at neutral pH, folds into a seventh α-helix that enters the binding cavity [17]. It has been suggested that this mechanism would push the ligand out of the binding pocket in the proximity of the olfactory receptor. The conformational change would be triggered by the lower pH present on the membrane surface. Although such conformational change has been clearly observed with several OBPs [18], the mechanism of ligand release has been questioned [19]. With other OBPs, the C-terminus is not long enough to make an additional helix and enter the binding cavity, but forms a sort of lid that covers the binding pocket, possibly contributing to the binding of specific ligands [20], [21].

A different mechanism has been demonstrated to be active with *Drosophila* LUSH. Upon binding its specific ligand vaccenyl acetate, this protein undergoes a conformational change involving a single amino acid residue. Such small change is enough to trigger binding of the complex to the specific olfactory receptor, thus generating the electrophysiological response of the neuron. This mechanism has been elegantly demonstrated showing that a mutant of LUSH mimicking the conformation this protein assumes in the complex with the pheromone is enough to produce an electrophysiological signal from the corresponding neuron even in the absence of the pheromone [22].

In this paper we have investigated the binding properties of OBP7 in two *Helicoverpa* moth species, a protein specifically expressed in antennae and likely involved in pheromone recognition. We also provide evidence that, unlike other OBPs of Lepidoptera, this protein does not undergo conformational changes at the C-terminus, that instead is involved in binding the pheromone components through the action of a specific lysine residue.

## Results and Discussion

### Preparation of Recombinant Proteins and Western Blot Experiments

The two sibling Lepidopteran species *Helicoverpa armigera* and *Helicoverpa assulta* share the same two major pheromone components, Z-11-hexadecenal (Z11-16:Ald) and Z-9-hexadecenal (Z9-16:Ald), but in ratios of 100∶2 and 6∶100, respectively [23]. Both species are endowed with several genes encoding proteins of the OBP family. In *H. armigera* these have been grouped into 3 PBPs (pheromone-binding proteins), 2 GOBPs (general odorants binding proteins) and 17 OBPs, while in *H. assulta* a lower number of genes have been annotated. Such classification of Lepidopteran OBPs was first suggested on the evidence that PBPs bind specific pheromones and are often male exclusive or more abundant, while GOBPs, being equally expressed in both sexes could be dedicated to binding general plant odours [24], [25]. Recent data have shown, however, that the picture could be more complex as at least GOBPs might be involved in detection of pheromones [26].

**Figure 1:**
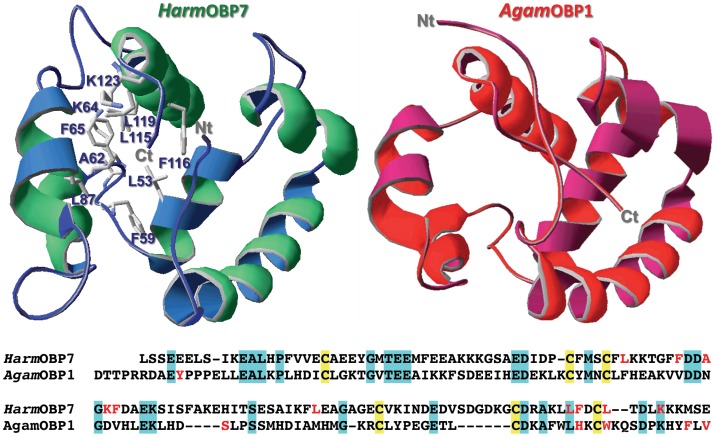
Three-dimensional model of *Harm*OBP7-wt. The model was built on the crystal structure of OBP1 of *Anopheles gambiae* (acc. 3 n7 hB [28]). In the alignment of the two proteins, cysteines are highlighted in yellow, common residues in light blue. Residues lining the binding pockets are in red font and are labelled in the model of *Harm*OBP7. Three phenylalanine and four leucine residue are inside the binding pocket, in agreement with the affinity of this protein for hydrophobic linear compounds. The C-terminus is shorter with respect to those of PBPs of this species and other Lepidoptera and cannot enter the binding site. Instead, it provides a sort of lid, which proved to be important for best fitting of some ligands. In particular, lysine 123 contributes to stabilising the binding of the two aldehyde pheromone components and structurally similar compounds, probably through Schiff bases or hydrogen bonds.

In the course of our study, aimed at characterising the OBPs of *H. armigera* and *H. assulta*, we have observed that the expression of OBP7 is antenna-specific and decided to further investigate the ligand-binding properties of this protein. The sequence of *Harm*OBP7 is reported in Figure 1 and presents the motif of 6 cysteines typical of “classic” OBPs. Its orthologue in the sibling species *H. assulta* (*Hass*OBP7) is nearly identical, except for two amino acid substitutions (K96Q and M126L).

**Figure 2:**
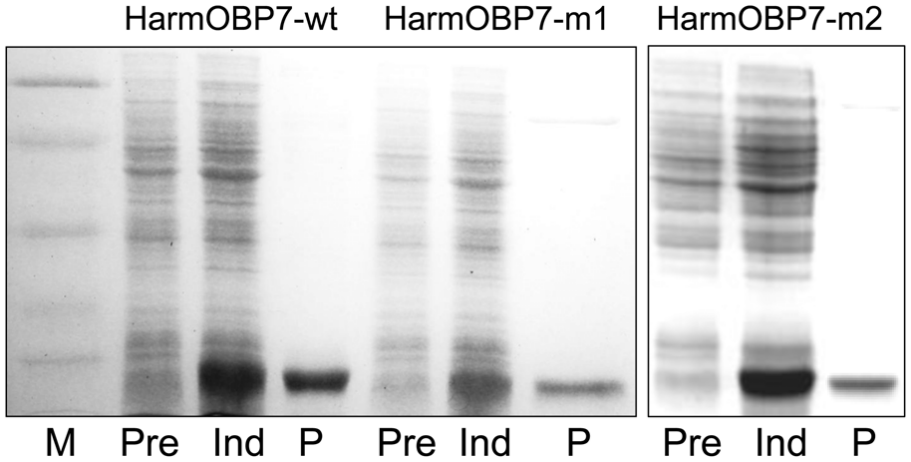
Expression and purification of the OBP7 of *H. armigera*, as well as of two mutants. *Harm*OBP7-m1 lacks the last 6 amino acids with respect to the wild type, while HarmOBP7-m2 bears the single amino acid substitution K123M. All three proteins were expressed in good yields (about 40 mg/L) and in soluble form. Purification was accomplished by anion-exchange chromatography on DE-52, followed by a second step on QFF. Molecular weight markers are, from the top, 66, 45, 29, 20 and 14 kDa.

**Figure 3:**
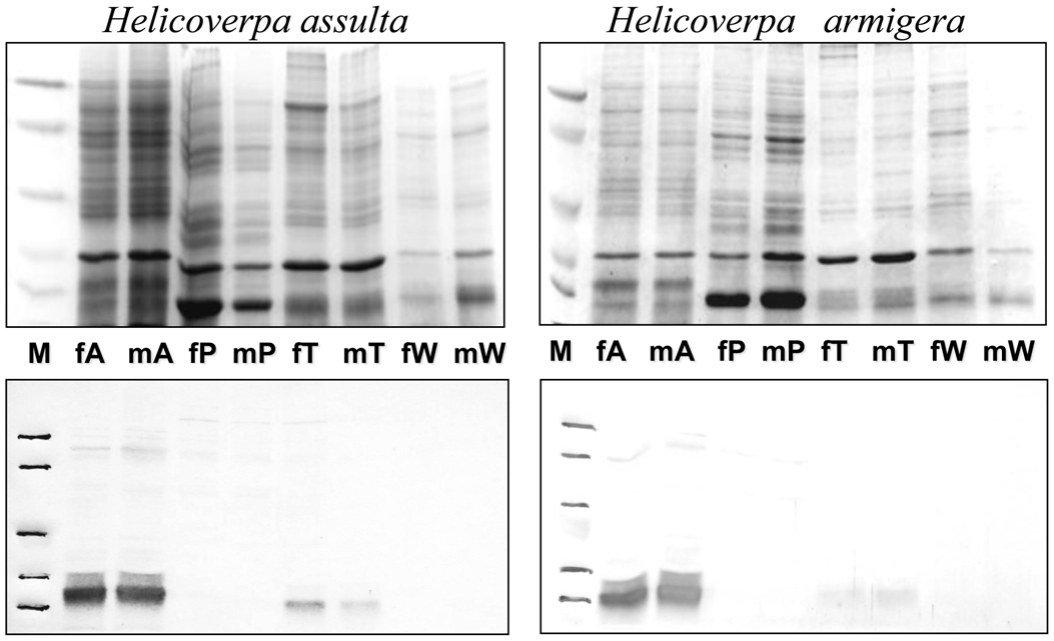
SDS-PAGE and Western blot of extracts from parts of the body of adults *H. armigera* and *H. assulta*. Upper panels: SDS-PAGE; lower panels: Western blot. (A); antennae, (P): proboscis, (T): tarsi, (W): wings of males (m) and females (f). The expression of OBP7 is limited to antennae with no significant differences between sexes or species. A weak staining in the extract of tarsi might indicate low levels of expression of OBP7 in such organ or cross-reactivity with other OBPs. Molecular weight markers (**M**) are as in Figure 1.

Therefore, we have expressed *Harm*OBP7 and a mutant lacking the last 6 residues (*Harm*OBP7-m1) in a bacterial system. Both proteins were obtained in high yields and purified adopting standard protocols through chromatographic steps on anion exchange resins DE-52 and QFF. Figure 2 reports the electrophoretic analysis of bacterial pellets and fractions of purified proteins for the wild-type (*Harm*OBP7-wt) and the mutant (*Harm*OBP7-m1), as well as for a second mutant (*Harm*OBP7-m2) that was later designed and synthesised based on the results described below. Polyclonal antisera, prepared against the purified recombinant *Harm*OBP7-wt were used to map the expression of the protein in different parts of the body in the two species *H. armigera* and *H. assulta*. In agreement with our previous information, we found that OBP7 is almost specifically expressed in the antennae of both species without significant differences between sexes (Figure 3). A barely detectable staining of a band in the tarsi extract could indicate low expression of OBP7 in this organ or cross-reaction with another structurally similar protein.

### Ligand-binding Assays

**Figure 4:**
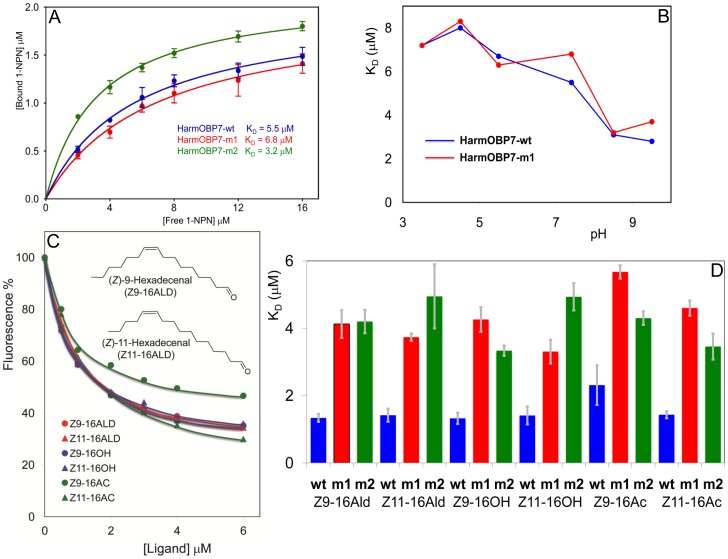
Binding of 1-NPN and pheromone and analogues to *Harm*OBP7 and its mutants. (**A**) Binding curves of 1-NPN (structure shown) to the three proteins. 2 µM solutions of each protein in Tris were titrated with 1 mM solution of 1-NPN in methanol to final concentrations of 2–16 µM. The data, averages of three replicates, were analysed using Prism software and indicated the presence of a single binding site. Dissociation constants were 5.5 µM for *Harm*OBP7-wt (SD 0.63), 6.8 µM for *Harm*OBP7-m1 (SD 1.1) and 3.2 µM for *Harm*OBP7-m2 (SD 0.22). (**B**) Effect of pH on the dissociation constants of the complexes with 1-NPN of *Harm*OBP-wt (circles) and its C-truncated mutant *Harm*OBP-m1 (triangles). Both proteins show maximum of activity at neutral and basic pH, without significant differences between wild type and mutant. The data are averages of three replicates. (**C**) Affinities of *Harm*OBP7-wt to pheromone components and their analogues. The structures of the two pheromone aldehydes are shown with their names. Analogues are the corresponding alcohols, indicated with the suffix OH and acetates, indicated with the suffix AC. Solutions of 2 µM proteins and 2 µM 1-NPN were titrated with 1 mM solution of each ligand in methanol to final concentrations of 0.5 to 6 µM. The figure reports averages of three replicates. (**D**) Dissociation constants of complexes between HarmOBP7-wt and its two mutants (*Harm*OBP7-m1 and *Harm*OBP7-m2) and the two aldehyde pheromone components, as well as structurally related alcohols and acetates. Both *Harm*OBP7-m1 and *Harm*OBP7-m2 bind all the compounds with lower affinity with respect to the wild type, indicating that the C-terminus, specifically Lys123, is involved in binding these ligands. Data are averages of three replicates. Standard errors are reported.

We then investigated the affinity of the fluorescent probe 1-NPN (N-phenyl-1-naphthylamine) to both proteins over a pH range from 3.5 to 9.5. Figure 4 reports the binding curves at pH 7.4 (Figure 4A) and the affinities of both proteins at different pH values (Figure 4B). In acidic media, down to pH 3.5, we could not observe significant loss of binding activity, while a marked increase in affinity was measured in basic conditions. Moreover, the lack of the C-terminal segment did not affect the performance of the protein over the entire pH range. This means that the conformational change reported for the PBP1 of *B. mori*, where the formation of the seventh α-helix was observed at pH 4–5 [17], [18], does not occur in our protein. Indeed, when examining the sequence of *Harm*OBP7 we could not expect such conformational change, as the C-terminus in this protein is 15 amino acid shorter than that of BmorPBP1, which is instead of the same length as in the three PBPs of *Helicoverpa* species [27]. In other OBPs of this sub-group (i.e. with a shorter C-terminus) it has been observed that the last segment, rather than entering the binding pocket, acts as a sort of lid and possibly becomes part of the binding cavity [20], [21].

Next we have performed a series of binding experiments with the wild type protein in order to define its specificity with particular reference to the pheromone components and structurally related molecules. The competitive binding curves are reported in Figure 4C, while the calculated dissociation constants are listed in Table 1. Overall, the behaviour of *Harm*OBP7-wt is similar to that of the three PBPs of the same species, reported in a previous paper [27], suggesting that this protein might contribute, together with the PBPs to detection and recognition of the specific pheromone blend. As verified with PBPs, *Harm*OBP7 also showed good affinity to the alcohols and acetates structurally related to the two pheromone components.

**Table 1:** Values of [IC]_50_ and calculated dissociation constants (µM) for the complexes between HarmOBP7-wt and various ligands.

Ligand	[IC]_50_	K_D_	Ligand	[IC]_50_	K_D_
Undecanal	10.7	8.1	2-Methylnaphthalene	17	12.9
Dodecanal	10.3	7.8	Cyclamen aldehyde	20	15.1
Tridecanal	4.9	3.7	4-Phenylanisole	18	13.6
Tetradecanal	3.2	2.4	p-tert-Butylbenzophenone	4.5	3.4
Heptadecanal	9.1	6.9	Methyl cinnamate	24	18.1
1-Decanol	20	15.1	Benzaldehyde	10.8	8.2
1-Dodecanol	>25		p-Isopropylbenzaldehyde	10.5	7.9
1-Tridecanol	4.6	3.5	3,7-Dimethyloctylbenzoate	3.5	2.7
1-Tetradecanol	6.7	5.1	Butyl cinnamate	9.6	7.3
1-Pentadecanol	>25		Butyl p-tert-butyl benzoate	6.9	5.2
1-Hexadecanol	20	15.1	α-Amylcinnamaldehyde	5.5	4.2
1-Octadecanol	>25		Coniferyl aldehyde	12.7	9.6
Ethyl decanoate	18	13.6	4-Phenylbutyrric acid	25	18.9
Ethyl laurate	4.3	3.3	Cinnamic acid	>25	
Ethyl myristate	4.7	3.5	2-Methoxycinnamaldehyde	14.7	11.1
Ethyl palmitate	12.6	12.6	Methyl cinnamate	22	16.7
Dodecyl acetate	5.2	3.9	Homovanillic acid	22	16.7
2-Dodecanone	18	13.6			
2-Tridecanone	9.6	7.3			

Values of [IC]_50_ larger than 16 have been extrapolated from the competitive binding curves.

**Figure 5:**
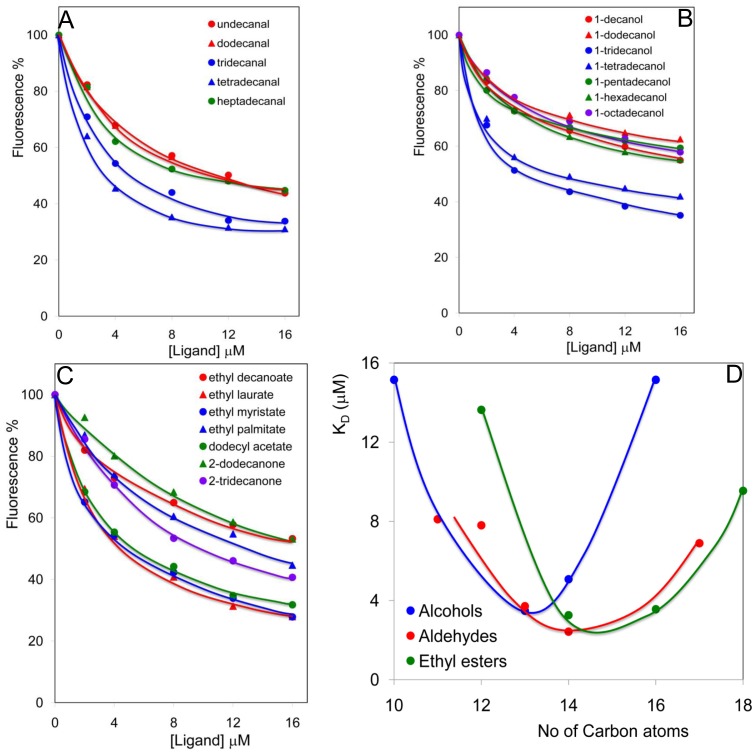
Affinities of *Harm*OBP7-wt to series of linear saturated primary alcohols, aldehydes and ethyl esters. (**A–C**) Displacement curves obtained with compounds of the three series. Solutions of 2 µM proteins and 2 µM 1-NPN were titrated with 1 mM solution of each ligand in methanol to final concentrations of 0.5 to 6 µM. (**D**) Dissociation constants as function of chain length. Best affinities are observed with linear compounds of 13–14 carbon atoms.

In a second series of experiments, we tested aldehydes, alcohols and esters of various length, to define the best size of ligands for a good fitting. In all three groups of molecules, a minimum in the values of dissociation constant is observed with lengths of 13–14 carbon atoms (Figure 5). This is in agreement with the high affinity measured with the pheromone components and their analogues that, although being longer molecules (16 carbon atoms) present a bent conformation due to the *cis* double bond, which therefore results in compacting the shape of the molecules.

We have also tested a number of aromatic molecules and found several of them to be good ligands for *Harm*OBP7. The data relative to all the tested ligands are listed in Table 1. Binding of linear compounds and of aromatic derivatives by the same protein is not surprising, as the *cis* double bond present in the pheromone components and their analogues forces the carbon chain into a bent configuration that can mimick a ring in the region around the unsaturation. In the model of *Harm*OBP7 (Figure 1), built on the crystal structure of OBP1 of *Anopheles gambiae* (acc. 3 n7 hB, [28]), also reported in the same figure, we have observed that three phenylalanine residues are lining the binding pocket, likely interacting with the benzene ring of the aromatic ligands or the *cis* unsaturation of the pheromone components. On the other hand, it is known that OBPs are not highly specific and that the performance of the olfactory system as a whole is based on a combinatorial code: sensors with broad spectra of response can be activated by several semiochemicals, while each odorant molecule can stimulate several receptors. This picture has been verified at the level of olfactory receptors and very likely also OBPs work with a similar mechanism. Moreover, we should not forget that several ligands might bind the protein without being able to trigger the conformational change necessary for activating the corresponding olfactory receptors.

Finally, we measured binding affinity of the pheromone components and their analogues to the mutant *Harm*OBP7-m1 (Figure 4D). Unlike 1-NPN, whose binding was not affected by the lack of the C-terminal segment, the pheromone molecules, as well as their analogues, showed significantly poorer affinities to the mutant with respect to the wild type. This suggests that the C-terminus, acting as a lid on the binding pocket, contributes to the stability of the complex with such molecules, but is not involved in binding 1-NPN.

### Design and Synthesis of HarmOBP7-m2

To identify the amino acid residue involved in binding the pheromone molecules, we decided to prepare a second mutant by replacing one of the lysines, possible candidates for forming a Schiff base with the aldehyde group of the pheromone components or hydrogen bonds with other ligands, such as alcohols or esters with another residue. We reasoned that Lys124 could establish an electrostatic interaction with Asp121, and analogously Lys 125 could be linked to Glu128. This would leave Lys123 free to interact with the ligand.

Therefore, in the second mutant, *Harm*OBP7-m2, we substituted Lys123 with a methionine. Such mutation replaces the linear four carbon atom chain of lysine with a linear chain of about the same length, but without the reactive amino group. The protein was expressed in bacteria, purified (Figure 2) and utilised in binding experiments with the pheromone components and their analogues.

This second mutant binds 1-NPN with a dissociation constant of 3.2 µM, slightly better than the wild type, indicating that the mutation did not significantly affect the binding and that Lys123 is not involved in binding 1-NPN (Figure 4A). However, binding of pheromone components, as well as their analogues to *Harm*OBP7-m2 was much weaker than to the wild type and comparable to the strength observed with the first mutant (Figure 4D). This reasonably indicates that Lys123 is involved in binding the aldehyde pheromone components, as well as their related alcohols and acetates. The results also confirm the observation, reported with other OBPs [29], [30], that often different ligands might bind equally well to a protein, but with different orientations in the binding pocket, thus involving different residues in the interactions. From these results we can conclude that Lys123, although being involved in binding the long-chain aldehydes, alcohols and acetates is not strictly required for the binding, and that such compounds interact with OBP7 with their hydrophobic chain inside the binding pocket and the functional group on the mout of the cavity. A similar behaviour was obsterved with the human OBP, that presents good affinities for medium-length aldehydes, despite being a lipcalin, therefore structurally different from insect OBPs. Also in that case, a lysine at the entrance of the binding cavity was found to be responsible for stabilising the interaction with aldehydes by forming a hydrogen bond [31].

The specific expression of OBP7 in the antennae and its binding properties, similar to the PBPs of the same species [27] might indicate that this protein is also involved in the correct identification of the specific pheromone blend. Even assuming that PBPs might be primarily responsible for detecting the pheromone components, other proteins, such as this OBP7 and likely members of the GOBP sub-group, could contribute to a more complex, and therefore more reliable, olfactory map. However, binding specificities are not sufficient to predict the response of the olfactory system, even if limited at the level of OBPs. In fact, it is now accepted that the formation of a complex between protein and ligand does not necessarily lead to activation of the protein, just in the same way that not every compound binding a G protein-coupled receptor is also a likely agonist. Moreover, olfactory receptors are obligatory partners in the chain of events leading from ligand binding to activation of specific glomeruli in the antennal lobe.

## Materials and Methods

This study was carried out in strict accordance with the recommendations in the Guide for the Care and Use of Laboratory Animals of the Chinese Academy of Sciences. The protocol was approved by the Committee on the Ethics of Animal Experiments of Institute of Zoology, the Chinese Academy of Sciences.

### Insects

*H. armigera* and *H. assulta* were collected as larvae from Zhengzhou, Henan, China. The larvae were reared in the laboratory on artificial diet, the main components of which were wheat germ and tomato paste. Rearing took place at a temperature of 27±1°C with a photoperiod of 16 h:8 h, L:D. Pupae were sexed and males and females were put into separate cages for eclosion. Adults were fed with 10% honey solution.

### Reagents

All enzymes were from New England Biolabs. Oligonucleotides were custom synthesised at SinoGenoMax, Beijing, China. All other chemicals were purchased from Sigma-Aldrich and were of reagent grade, except selected compounds used in binding assays, which were prepared along with conventional synthetic routes.

### RNA Extraction and cDNA Synthesis

Total RNA was extracted from TRI® Reagent (Invitrogen), following the manufacturer’s protocol. cDNA was prepared from total RNA by reverse transcription, using 200 units of M-MLV Reverse Transcriptase (Promega) and 0.5 µg of an oligo-dT primer in a 25 µl total volume. The mixture also contained 0.5 mM of each dNTP (TaKaRa), and 5 µL reaction buffer. The reaction mixture was incubated at 42°C for 60 min and the product was directly used for PCR amplification or stored at −20°C.

### Polymerase Chain Reaction

Aliquots of 1 µL of crude cDNA were amplified in a Bio-Rad Gene CyclerTM thermocycler, using 2.5 units of Thermus aquaticus DNA polymerase (TaKaRa), 1 mM of each dNTP (TaKaRa), 1 µM of each PCR primer and 2.5 µL reaction buffer.

3′ends of the OBP7 was amplified by using SMARTer RACE cDNA Amplification Kit (Clontech) following the kit instructions. Specific primer for 3′RACE was CGCAGAGGAATACGGAATGACAGA. For the recombinant *Harm*OBP7, at the 5′ end we used a specific primer encoding the first six amino acids of the mature protein preceded by an Nde I restriction site. At the 3′ end, the primer contained the sequence encoding the last six amino acids, followed by a stop codon and an EcoR I restriction site. After a first denaturation step at 95°C for 5 min., we performed 35 amplification cycles (1 min. at 95°C, 30 sec. at 55°C, 1 min. at 72°C) followed by a final step of 10 min. at 72°C.

### Cloning and Sequencing

The crude PCR products were ligated into a pGEM (Promega) vector without further purification, using a 1∶5 (plasmid:insert) molar ratio and incubating the mixture overnight at 4°C. After transformation of *E. coli* Top10 competent cells with the ligation products, positive colonies were selected by PCR using the plasmid’s primers SP6 and T7, grown in LB/ampicillin medium and custom sequenced at SinoGeno Max, Beijing, China.

### Cloning in Expression Vectors

pGEM plasmid containing the sequence encoding the mature protein, flanked by the two restriction sites, was digested with Nde I and EcoR I restriction enzymes for two hours at 37°C and the digestion product was separated on agarose gel. The obtained fragments was purified from gel using TaKaRa MiniBest Plasmid Purification Kit (TaKaRa) and ligated into the expression vector pET30b, previously linearized with the same enzymes. The resulting plasmid was sequenced and shown to encode the mature protein.

### Preparation of Mutants

The truncated form of the *Harm*OBP7 was obtained by inserting a stop codon after Leu122. The pET30b plasmid containing the target sequence was amplified using T7 as the forward primer and an oligonucleotide encoding amino acids 119–127, where the codon for residue 123 had been replaced by a stop codon as the reverse primer.

The mutant K123M was prepared along with the same procedure, but using the sequence GCTCATTTTCTTCTAAAGGTCCGTCAA as reverse primer.

Conditions were as follows: initial denaturation at 95°C for 5 min, followed by 9 cycles of 95°C for 30 sec, 50°C for 1 min and 68°C for 1 min, followed by 9 cycles of 95°C for 30 sec and 68°C for 6 min, and final extension at 68°C for 16 min. The crude PCR product was digested with DpnI for 3 hours at 37°C and used to transform *E. coli* cells. Expression and purification was performed as described for the wild-type proteins.

### Preparation of the Protein

For expression of the recombinant *Harm*OBP7-wt and mutants, pET-30b vectors containing the sequences encoding the mature proteins were used to transform BL21 *E. coli* cells. Protein expression was induced by addition of IPTG to a final concentration of 0.4 mM when the culture had reached a value of O.D.600 = 0.8. Cells were grown for further 2 hours at 37°C, then harvested by centrifugation and sonicated. After centrifugation, all three proteins were present in the supernatant. The concentrations of the proteins were about 40 mg/L. OBP7 was not found in inclusion bodies. Purification of the proteins was accomplished by combinations of chromatographic steps on anion exchange resins, such as DE-52 (Whatman) and QFF, along with standard protocols previously adopted for other odorant-binding proteins [32], [33].

### Preparation of the Antiserum

An antiserum against *Harm*OBP7-wt was obtained by injecting a rabbit subcutaneously with 300 µg of recombinant protein, followed by three additional injections of 200 µg after 7 days each time. The protein was emulsified with an equal volume of Freund’s complete adjuvant for the first injection and incomplete adjuvant for further injections. The rabbit was bled one week after the last injection and the serum was used without further purification. The rabbit was housed in a large cage, at constant temperature, and all operations were performed according to ethical guidelines to minimize pain and discomfort to the animal.

### Western Blot Analysis

After electrophoretic separation under 14% sodium dodecyl sulphate polyacrylamide gel electrophoresis (SDS-PAGE), duplicate gels were stained with 0.1% Coomassie blue R250 in 10% acetic acid, 20% ethanol or electroblotted on Trans-Blot nitrocellulose membrane (Bio-Rad Lab) by the procedure of Kyhse-Andersen [34]. After treatment with 2% powdered skimmed milk/Tris overnight, the membrane was incubated with the crude antiserum against the protein at a dilution of 1∶500 (2 h), then with goat anti-(rabbit IgG) horseradish peroxidase conjugate (dilution 1∶1000; 1 h). Immunoreacting bands were detected by treatment with 4-chloro-1-naphthol and hydrogen peroxide.

### Fluorescence Measurements

Emission fluorescence spectra were recorded on a Hitachi F-4500 at 25°C in a right angle configuration, with a 1 cm light path quartz cuvette and 5 nm slits for both excitation and emission. The protein was dissolved in 50 mM Tris-HCl buffer, pH 7.4, while ligands were added as 1 mM methanol solutions.

### Fluorescence Binding Assays

To measure the affinity of the fluorescent ligand 1-NPN to *Harm*OBP7-wt and mutants, a 2 µM solution of the protein in 50 mM Tris-HCl, pH 7.4, was titrated with aliquots of 1 mM ligand in methanol to final concentrations of 2–16 µM. The probe was excited at 337 nm and emission spectra were recorded between 380 and 450 nm. The affinities of other ligands were measured in competitive binding assays, where a solution of the protein and 1-NPN, both at the concentration of 2 µM was titrated with 1 mM methanol solutions of each competitor over concentration ranges of 0.5–6 µM or 2–16 µM, depending on the ligand. Dissociation constant for 1-NPN and the stoichiometry of binding was obtained by processing the data with Prism software. Dissociation constants of the competitors were calculated from the corresponding [IC]50 values (concentrations of ligands halving the initial fluorescence value of 1-NPN), using the equation: KD = [IC50]/1+[1-NPN]/K1-NPN, [1-NPN] being the free concentration of 1-NPN and K1-NPN being the dissociation constant of the complex protein/1-NPN.

### Molecular Modelling

A three-dimensional model of *Harm*OBP7-wt was generated using the on-line programme SWISS MODEL [35]–[37]. The structure of OBP1 of *Anopheles gambiae*, acc. No. 3 n7 hB [28], was used as a template (identity between the two proteins: 23.3%). Models were displayed using the SwissPdb Viewer programme “Deep-View” [36] (http://www.expasy.org/spdbv/).

## References

[1] Sandler BH, Nikonova L, Leal WS, Clardy J (2000) Sexual attraction in the silkworm moth: structure of the pheromone-binding protein-bombykol complex. Chem Biol 7: 143–151. 10662696 10.1016/s1074-5521(00)00078-8

[2] Bianchet MA, Bains G, Pelosi P, Pevsner J, Snyder SH, et al (1996) The three dimensional structure of bovine odorant-binding protein and its mechanism of odor recognition. Nat Struct Biol 3: 934–939. 8901871 10.1038/nsb1196-934

[3] Tegoni M, Ramoni R, Bignetti E, Spinelli S, Cambillau C (1996) Domain swapping creates a third putative combining site in bovine odorant binding protein dimmer. Nat Struct Biol 3: 863–867. 8836103 10.1038/nsb1096-863

[4] Pelosi P, Maida R (1990) Odorant binding proteins in vertebrates and insects: similarities and possible common function. Chem Senses 15: 205–215.

[5] Pelosi P (1996) Perireceptor events in olfaction. J Neurobiol 30: 3–19. 8727979 10.1002/(SICI)1097-4695(199605)30:1<3::AID-NEU2>3.0.CO;2-A

[6] Vogt RG, Riddiford LM (1981) Pheromone binding and inactivation by moth antennae. Nature 293: 161–163. 18074618 10.1038/293161a0

[7] Vogt RG (2005) Molecular basis of pheromone detection in insects. In: Gilbert LI, Iatrou K, Gill S, eds. Comprehensive Insect Physiology, Biochemistry, Pharmacology and Molecular Biology. Endocrinology Vol 3. London: Elsevier. 753–804.

[8] Pelosi P, Pisanelli AM, Baldaccini NE, Gagliardo A (1981) Binding of [3H]-2-sobutyl-3-methoxypyrazine to cow olfactory mucosa. Chem Senses 6: 77–85.

[9] Pelosi P, Baldaccini NE, Pisanelli AM (1982) Identification of a specific olfactory receptor for 2-isobutyl-3-methoxypyrazine. Biochem J 201: 245–248. 7082286 10.1042/bj2010245 PMC1163633

[10] Pelosi P, Zhou J-J, Ban LP, Calvello M (2006) Soluble proteins in insect chemical communication. Cell Mol Life Sci 631: 658–1676. 10.1007/s00018-005-5607-0 PMC11136032 16786224

[11] Forstner M, Breer H, Krieger J (2009) A receptor and binding protein interplay in the detection of a distinct pheromone component in the silkmoth *Antheraea polyphemus.* . Int J Biol Sci 5: 745–757. 20011135 10.7150/ijbs.5.745 PMC2793307

[12] Xu PX, Atkinson R, Jones DN, Smith DP (2005) *Drosophila* OBP LUSH is required for activity of pheromone-sensitive neurons. Neuron 45: 193–200. 15664171 10.1016/j.neuron.2004.12.031

[13] Matsuo T, Sugaya S, Yasukawa J, Aigaki T, Fuyama Y (2007) Odorant-binding proteins OBP57d and OBP57e affect taste perception and host-plant preference in *Drosophila sechellia* . PLoS Biol 5: e118. 17456006 10.1371/journal.pbio.0050118 PMC1854911

[14] Swarup S, Williams TI, Anholt RRH (2011) Functional dissection of odorant binding protein genes in *Drosophila melanogaster* . Genes Brain Behav 10: 648–657. 21605338 10.1111/j.1601-183X.2011.00704.x PMC3150612

[15] Qiao HL, Tuccori E, He XL, Gazzano A, Field LM, et al (2009) Discrimination of alarm pheromone (E)-β-farnesene by aphid odorant-binding proteins. Insect Biochem Mol Biol 39: 414–419. 19328854 10.1016/j.ibmb.2009.03.004

[16] Sun YF, De Biasio F, Qiao HL, Iovinella I, Yang SX, et al (2012) Two odorant-binding proteins mediate the behavioural response of aphids to the alarm pheromone (E)-β-farnesene and structural analogues. PLoS One 7: e32759. 22427877 10.1371/journal.pone.0032759 PMC3299684

[17] Horst R, Damberger F, Luginbuhl P, Guntert P, Peng G, et al (2001) NMR structure reveals intramolecular regulation mechanism for pheromone binding and release. Proc Natl Acad Sci USA. 98: 14374–14379. 10.1073/pnas.251532998 PMC64689 11724947

[18] Damberger FF, Ishida Y, Leal WS, Wuthrich K (2007) Structural basis of ligand binding and release in insect pheromone-binding proteins: NMR structure of *Antheraea polyphemus* PBP1 at pH 4.5. J Mol Biol 373: 811–819. 17884092 10.1016/j.jmb.2007.07.078

[19] Gong Y, Pace TCS, Castillo C, Bohne C, O’Neill MA, et al (2009) Ligand-interaction kinetics of the pheromone-binding protein from the gypsy moth *L. dispar*: insights into the mechanism of binding and release. Chem Biol 16: 162–172. 19246007 10.1016/j.chembiol.2009.01.005

[20] Tegoni M, Campanacci V, Cambillau C (2004) Structural aspects of sexual attraction and chemical communication in insects. Trends Biochem Sci 29: 257–264. 15130562 10.1016/j.tibs.2004.03.003

[21] Leite NR, Krogh R, Xu W, Ishida Y, Iulek J, et al (2009) Structure of an odorant-binding protein from the mosquito *Aedes aegypti* suggests a binding pocket covered by a pH-sensitive “lid”. PLoS One 4: e8006. 19956631 10.1371/journal.pone.0008006 PMC2778553

[22] Laughlin JD, Ha TS, Jones DNM, Smith DP (2008) Activation of pheromone-sensitive neurons is mediated by conformational activation of pheromone-binding protein. Cell 133: 1255–1265. 18585358 10.1016/j.cell.2008.04.046 PMC4397981

[23] Wang HL, Zhao CH, Wang CZ (2005) Comparative study of sex pheromone composition and biosynthesis in *Helicoverpa armigera*, *H. assulta* and their hybrid. Insect Biochem Mol Biol 35: 575–583. 15857763 10.1016/j.ibmb.2005.01.018

[24] Vogt RG, Prestwich GD, Lerner M (1991) Odorant-Binding-Protein subfamilies associate with distinct classes of olfactory receptor neurons in insects. J Neurobiol 22: 74–84. 2010751 10.1002/neu.480220108

[25] Vogt RG, Prestwich GD, Lerner MR (1991) Molecular cloning and sequencing of General-Odorant Binding Proteins GOBP1 and GOBP2 from Tobacco Hawk Moth *Manduca sexta*: comparisons with other insect OBPs and their signal peptides. J Neurosci 11: 2972–2984. 1719155 10.1523/JNEUROSCI.11-10-02972.1991 PMC6575436

[26] Zhou J-J, Robertson G, He X, Dufour S, Hooper AM (2009) Characterisation of *Bombyx mori* odorant-binding proteins reveals that a general odorant-binding protein discriminates between sex pheromone components. J Mol Biol 389: 529–545. 19371749 10.1016/j.jmb.2009.04.015

[27] Guo H, Huang LQ, Pelosi P, Wang CZ (2012) Three pheromone-binding proteins help segregation between two *Helicoverpa* species utilizing the same pheromone components. Insect Biochem Mol Biol 42: 708–716. 22750167 10.1016/j.ibmb.2012.06.004

[28] Wogulis M, Morgan T, Ishida Y, Leal WS, Wilson DK (2006) The crystal structure of an odorant binding protein from *Anopheles gambiae*: evidence for a common ligand release mechanism. Biochem Biophys Res Commun 339: 157–164. 16300742 10.1016/j.bbrc.2005.10.191

[29] Vincent F, Spinelli S, Ramoni R, Grolli S, Pelosi P, et al (2000) Complexes of porcine odorant binding protein with odorant molecules belonging to different chemical classes. J Mol Biol 300: 127–139. 10864504 10.1006/jmbi.2000.3820

[30] Spinelli S, Lagarde A, Iovinella I, Legrand P, Tegoni M, et al (2012) Crystal structure of *Apis mellifera* OBP14 a C-minus odorant-binding protein and its complexes with odorant molecules. Insect Biochem Mol Biol 42: 41–50. 22075131 10.1016/j.ibmb.2011.10.005

[31] Tcatchoff L, Nespoulous C, Pernollet JC, Briand L (2006) A single lysyl residue defines the binding specificity of a human odorant-binding protein for aldehydes. FEBS Lett 580: 2102–2108. 16546182 10.1016/j.febslet.2006.03.017

[32] Ban LP, Scaloni A, D’Ambrosio C, Zhang L, Yan YH, et al (2003) Biochemical characterisation and bacterial expression of an odorant-binding protein from *Locusta migratoria* . Cell Mol Life Sci 60: 390–400. 12678502 10.1007/s000180300032 PMC11138600

[33] Calvello M, Guerra N, Brandazza A, D’Ambrosio C, Scaloni A, et al (2003) Soluble proteins of chemical communication in the social wasp *Polistes dominulus* . Cell Mol Life Sci 60: 1933–1943. 14523553 10.1007/s00018-003-3186-5 PMC11138633

[34] Kyhse-Andersen J (1984) Electroblotting of multiple gels: a simple apparatus without buffer tank for rapid transfer of proteins from polyacrylamide to nitrocellulose. J Biochem Biophys Methods 10: 203–209. 6530509 10.1016/0165-022x(84)90040-x

[35] Arnold K, Bordoli L, Kopp J, Schwede T (2006) The SWISS-MODEL Workspace: A web-based environment for protein structure homology modelling. Bioinformatics 22: 195–201. 16301204 10.1093/bioinformatics/bti770

[36] Guex N, Peitsch MC (1997) SWISS-MODEL and the Swiss-Pdb Viewer: An environment for comparative protein modelling. Electrophoresis 18: 2714–2723. 9504803 10.1002/elps.1150181505

[37] Schwede T, Kopp J, Guex N, Peitsch MC (2003) SWISS-MODEL: an automated protein homology-modelling server. Nucleic Acids Res 31: 3381–3385. 12824332 10.1093/nar/gkg520 PMC168927

